# Which Kind of Provider’s Operation Volumes Matters? Associations between CABG Surgical Site Infection Risk and Hospital and Surgeon Operation Volumes among Medical Centers in Taiwan

**DOI:** 10.1371/journal.pone.0129178

**Published:** 2015-06-08

**Authors:** Tsung-Hsien Yu, Yu-Chi Tung, Kuo-Piao Chung

**Affiliations:** Institute of Health Policy and Management, National Taiwan University, Taipei, Taiwan; Providence VA Medical Center and Brown University, UNITED STATES

## Abstract

**Background:**

Volume-infection relationships have been examined for high-risk surgical procedures, but the conclusions remain controversial. The inconsistency might be due to inaccurate identification of cases of infection and different methods of categorizing service volumes. This study takes coronary artery bypass graft (CABG) surgical site infections (SSIs) as an example to examine whether a relationship exists between operation volumes and SSIs, when different SSIs case identification, definitions and categorization methods of operation volumes were implemented.

**Methods:**

A population-based cross-sectional multilevel study was conducted. A total of 7,007 patients who received CABG surgery between 2006 and 2008 from19 medical centers in Taiwan were recruited. SSIs associated with CABG surgery were identified using International Classification of Diseases, 9th Revision, Clinical Modification (ICD-9 CM) codes and a Classification and Regression Trees (CART) model. Two definitions of surgeon and hospital operation volumes were used: (1) the cumulative CABG operation volumes within the study period; and (2) the cumulative CABG operation volumes in the previous one year before each CABG surgery. Operation volumes were further treated in three different ways: (1) a continuous variable; (2) a categorical variable based on the quartile; and (3) a data-driven categorical variable based on k-means clustering algorithm. Furthermore, subgroup analysis for comorbidities was also conducted.

**Results:**

This study showed that hospital volumes were not significantly associated with SSIs, no matter which definitions or categorization methods of operation volume, or SSIs case identification approaches were used. On the contrary, the relationships between surgeon’s volumes varied. Most of the models demonstrated that the low-volume surgeons had higher risk than high-volume surgeons.

**Conclusion:**

Surgeon volumes were more important than hospital volumes in exploring the relationship between CABG operation volumes and SSIs in Taiwan. However, the relationships were not robust. Definitions and categorization methods of operation volume and correct identification of SSIs are important issues for future research.

## Introduction

The Luft *et al*. classical article published in 1979[1] aroused research interest in the volume-outcome relationship and triggered further research on several related topics. In the past decade, the outcome measures used in volume-outcome studies have gradually changed from mortality to other complications, such as surgical site infections (SSIs).

Although there are an enormous number of studies in the literature exploring the volume-outcome issue, the findings are not consistent. In the topic of volume-infection, the controversial findings can perhaps be attributed to two major issues. Firstly, the identification of infection cases might not have been accurate. Most studies analyzed the relationship between provider volumes and infection on the basis of claims data. [2–4] Researchers usually have identified infection cases through International Classification of Diseases, 9th Revision, Clinical Modification (ICD-9-CM) infection codes. In contrast to surveillance data, the use of administrative data can increase sample sizes due to reduced labor intensity and also facilitate the efficiency and standardization of case identifications. [5] However, several studies have indicated that the ICD-9-CM codes might be inappropriate for identifying such cases in claims data because of insufficient code list and coding bias. [2, 6–8] These problems might not only over- or under-estimate the number of infection cases, but also affect the validity of studies, especially in patient-level studies. [9, 10] The use of surrogate markers or development of identification models has been popular in the identification of cases of healthcare-associated infections since 2000. For example, Yu et al [11] used utilization of antibiotics (e.g. type, dose, second-line antibiotics), length of stay, and number of vessels obstructed etc., to develop the CART and other alternative models for the identification of cases of CABG SSIs, based on the National Health Insurance claims data and healthcare-associated infection surveillance data from two medical centers in Taiwan, and compared the performance between these models and the ICD-9 CM codes.

Secondly, the definition and categorization methods of service or operation volumes are not consistent. In the past, some studies calculated the service/ operation volumes within the study period, while others calculated the service/ operation volumes before the study period (e.g. in the previous one year). The latter definition might better reflect the provider’s level of experience at the time a patient received healthcare services than former one. [12]

Moreover, researchers usually categorized provider volumes using subjective methods. Many studies categorized service volumes into low-, medium- and high-volume groups in different ways [13–15], and some studies used a specific case number [16] or percentage [17] as the cutoff value for categorization. The heterogeneity of categorization methods might have produced different results.[12, 18, 19] Furthermore, the neglect of cluster characteristics of data, which should use hierarchical models, may result in biased estimation of the variation and also lead to incorrect conclusions.

SSIs following coronary artery bypass graft (CABG) procedures place a heavy burden on patients and healthcare systems. The total length of stay and expenditure for patients with SSIs after CABG surgery is significantly longer and higher than those without SSIs. [20, 21] In 2008, the Centers for Medicare & Medicaid of the United States of America implemented the “Never Event” policy, where hospitals would no longer receive higher payments for the additional costs associated with treating patients for certain healthcare-acquired infections, including those related to CABG.

In view of the accuracy of SSIs identification and the heterogeneity of definition and categorization methods, no existing studies have used different infection case identification nor definitions and categorization methods of operation volume simultaneously to explore the relationship between operation volumes and infection. The current study takes CABG SSIs as an example to examine whether a relationship exists between operation volumes and SSIs, given different SSI cases identification, operation volume definitions and categorization methods.

## Materials and Methods

### Study design

This retrospective and cross-sectional study adopted a multilevel design to examine the relationships between provider volumes and SSIs after adjusting for patient-, surgeon-, and hospital-level covariates.

### Data sources

We used data from the Taiwan National Health Insurance Research Database (NHIRD) from 2005 and 2008. The NHIRD, published by the Taiwan National Health Research Institute, includes all the original claims data and registration files for beneficiaries enrolled under the National Health Insurance (NHI) program. The database covers the 23 million Taiwanese enrollees (approximately 98% of the population) in the NHI program. It is a de-identified secondary database containing patient-level demographic and administrative information; however, treatment items are aggregated and without time-related and clinical information. The data is released for research purposes.

### Ethics Statement

The protocol for the study was approved by the Institutional Review Board of the National Taiwan University Hospital (protocol #201001027R). The dataset we used in this study was secondary data; all information was de-identified by data owners.

### Dependent variable: Surgical Site Infection Cases identification

In this study, we adopted the ICD-9-CM SSI codes (hereafter referred to as the ICD-9-CM based model) and the Classification and Regression Trees (CART) model, which was developed in our previous work [11] to identify SSI cases. As we mentioned above, the ICD-9-CM SSI codes were the most popular tool to identify the SSI cases in claims data. In the ICD-9-CM based model, SSI cases were divided into two categories: index hospitalization events and post-discharge events (i.e., SSIs that occurred within 1 year after discharge and required readmission to a hospital and/or the use of ambulatory services). Following Wu *et al* [13], this study adopted the secondary ICD-9-CM diagnosis codes for index hospitalization events (ICD-9-CM code: 996.03, 996.61, 996.72, and 998.5), and the primary and secondary diagnosis codes for post-discharge events (ICD-9-CM code: 038.0–038.4, 038.8, 038.9, 682.6, 682.9, 780.6, 790.7, 875.0, 875.1, 891.0, 891.1, 996.03, 996.61, 996.72, 998.3, and 998.5.) as the criteria for SSI identification, in order to avoid cases in which infection existed prior to hospitalization. If a case had an index hospitalization event or a post-discharge event, then he/ she will be identified as SSIs by the ICD-9-CM based model.

In the CART model, we adopted the type of antibiotics, dose of cefazolin, length of stay, and number of vessels obstructed (as a proxy indicator of duration of operation) as the parameters to identify the SSIs, according to our previous findings. [11] In our previous work, we used the 2005–2008 National Health Insurance claims data and healthcare-associated infection surveillance data from two medical centers for model development and model verification. Infection cases based on surveillance were identified by infection control personnel if the patient met the Taiwan CDC’s criteria, which are the same as those adopted in the U.S. CDC. They manually review medical records of all patients at risk for the specified healthcare-associated infection.

The classification algorithms, the multivariable regression model, and the data mining model were adopted to develop alternative models based on surrogate indicators to identify cases of CABG SSIs and to compare the performance among these models and the ICD-9-CM- based model. For the classification algorithms, researchers build up several criteria, and if a case satisfies (or exceeds) a specific number of criteria, then it will be identified as a case of infection. For the multivariable regression model, researchers usually calculated a risk score by the logistic regression model, and the optimal cutoff point was determined according to the resulting receiver operating characteristic curve.

Concerning the data mining approach, which is widely used for predicting and classifying objects, the characteristics are: automatic discovery of patterns, prediction of likely outcomes, creation of actionable information, and focus on large data sets and databases. The classification and regression tree (CART) model, which is the most popular approach as applied in our work, and the growing, stopping, and pruning of the tree were determined by Gini improvement measures. [22, 23] After referring to the literature and conferring with infectious disease specialists, we adopted the following seven parameters: type of antibiotic, doses of antibiotic, doses of cefazolin, use of second-line antibiotics, length of stay, and number of vessels obstructed. Additionally, cross-validation was also employed, where data from one medical center was used for model development, and another one was used for model validation.

The results of our previous work revealed that the CART model offered better performance than that of the other identification models or the ICD-9-CM based model, especially in the positive predictive value (>70%), which was only found to be 20% in the ICD-9-CM based model. (Table 1) The findings also implied that the CART was a decidedly better tool for identifying cases of SSI in the Taiwan National Health Insurance database. Therefore, this study also adopted the CART model for identifying CABG SSIs.

**Table 1:** Performance comparisons between ICD-9-CM-based and CART models for identifying CABG Surgical Site Infections.

	Sensitivity	Specificity	PPV	NPV	Accuracy
**Model development**					
ICD-9-CM SSI code	37.50%(9/24)	96.27%(956/993)	19.57%(9/46)	98.46%(956/971)	94.89%(965/1017)
CART	87.50%(21/24)	99.40%(987/993)	77.78%(21/27)	99.70%(987/990)	99.12%(1008/1017)
**Model validation**					
ICD-9-CM SSI code	35.29% (6/17)	96.98% (803/828)	19.35% (6/31)	98.65% (803/814)	95.74% (809/845)
CART	88.24% (15/17)	99.28% (822/828)	71.43% (15/21)	99.76% (822/824)	99.05% (838/845)

% (numerator/ denominator)

PPV: positive predictive value; NPV: negative predictive value.

Source: Yu *et al*., 2014(11)

To ensure homogeneity, current study analyzed 7,007 patients from 19 medical centers in Taiwan who underwent CABG surgery (ICD-9-CM procedure codes 36.1x–36.2x) between 2006 and 2008. CABG patients under the age of 18 years or over 85 years were excluded in this study. A total of 302 cases were identified as SSIs by ICD-9-CM based model, and a total of 107 cases were identified as SSIs by CART model.

### Independent and control variables

In this study, we used the following two definitions to define operation volumes: (1) the cumulative operation volumes by each surgeon and hospital within the study period, which was the most common definition in the literature; and (2) following Yasunaga et al.’s study, [24] cumulative operation volumes by each surgeon and hospital in the previous one year for each surgery. However, our data was skewed, which did not follow a normal distribution. Therefore, we conducted the log transformations on operation volumes.

The current work treated operation volumes in three different ways: (1) a continuous variable; (2) a categorical variable based on the first and the third quartile as cutoff points (the most common method to categorize service/ operation volumes) [25–28]; and (3) a data-driven categorical variable based on *k*-means clustering algorithm. This study categorized surgeon and hospital volumes into low, medium, and high volume groups by quartile method and k-means clustering algorithm.

In the quartile method, the cut-off value (transformed by logarithm) of the first quartile (<25%) for hospital volumes was 5.65, and the third quartile (>75%) was 6.43. In terms of surgeon volumes, the first quartile was 4.38, and the third was 5.35, when we used the cumulative operation volumes within the study period as the definition. While the definition changed, first quartile (<25%) for hospital volumes was 4.66, and the third quartile (>75%) was 5.31. In terms of surgeon volumes, the first quartile was 3.40, and the third was 4.32.

K-means clustering is an unsupervised machine-learning algorithm introduced by MacQueen in 1960s. This method is not only a simple and very reliable method in categorization/ classification, but is also recognized as one of the top 10 algorithms in data mining. [29] This method has often been applied in many fields. [30–32] Yu and his colleagues even applied it to define the quality of CABG care, and to explore the relationship among patient’s income status, the level of quality of care, and inpatient mortality. [33]

The main idea of this method is to partition observed data points into k non-overlapping clusters by minimizing the within-group sum of squares. Each point is assigned to the mean of its cluster using the Euclidian distance. Firstly, k cluster centers were randomly generated. Previous studies usually divided surgeons and hospitals into low-, medium-, and high-volume groups; therefore, we also predetermined the surgeon and hospital service volumes into 3 groups (k = 3). Then, participants were assigned to the cluster with the shortest distance to these cluster centers. Finally, the cluster centers were recomputed using the new cluster assignment and these steps would be iterated until convergence was achieved. [34]

**Fig 1:**
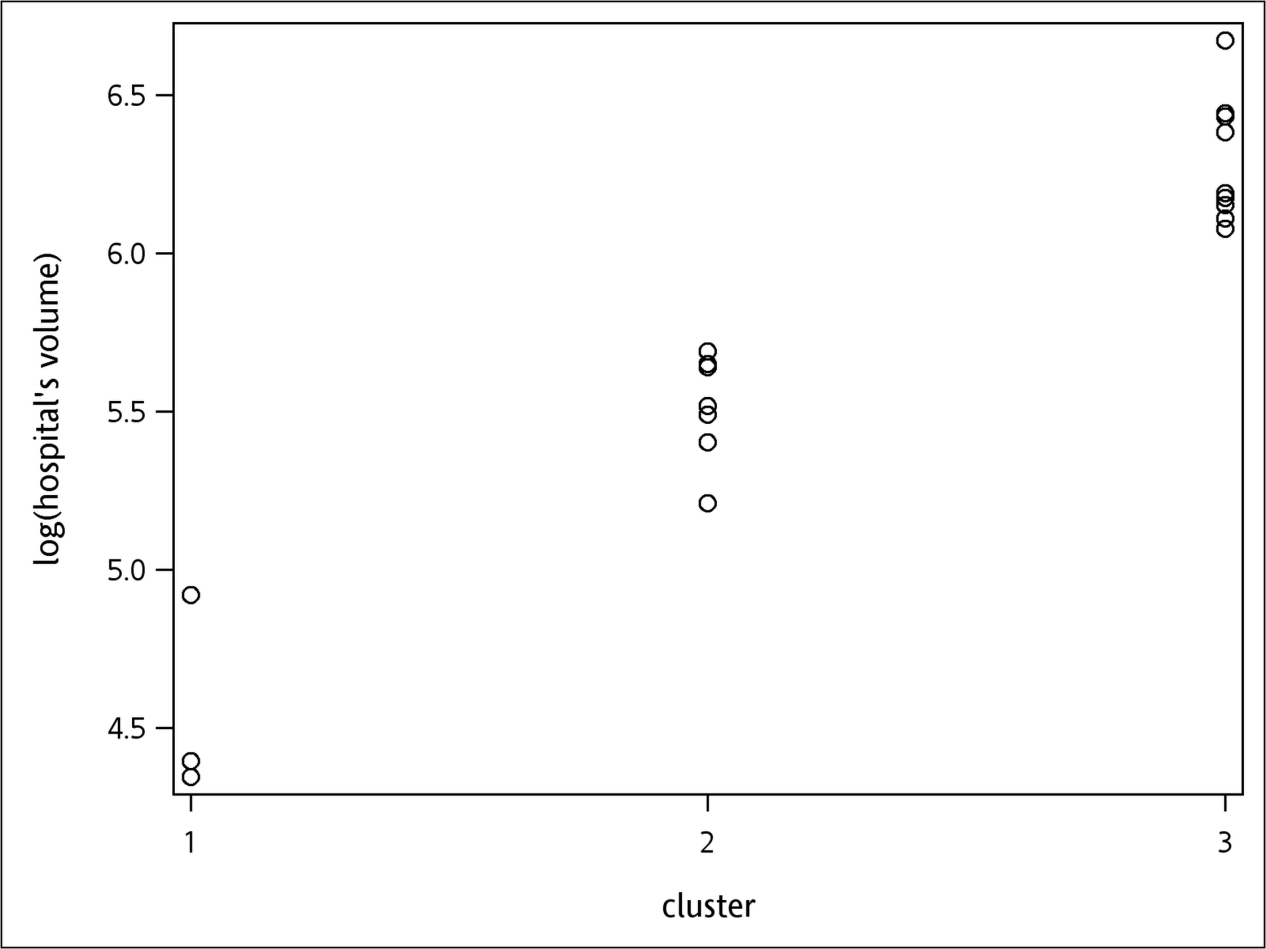
Scatter plot: hospital cumulative operation volumes within the study period.

**Fig 2:**
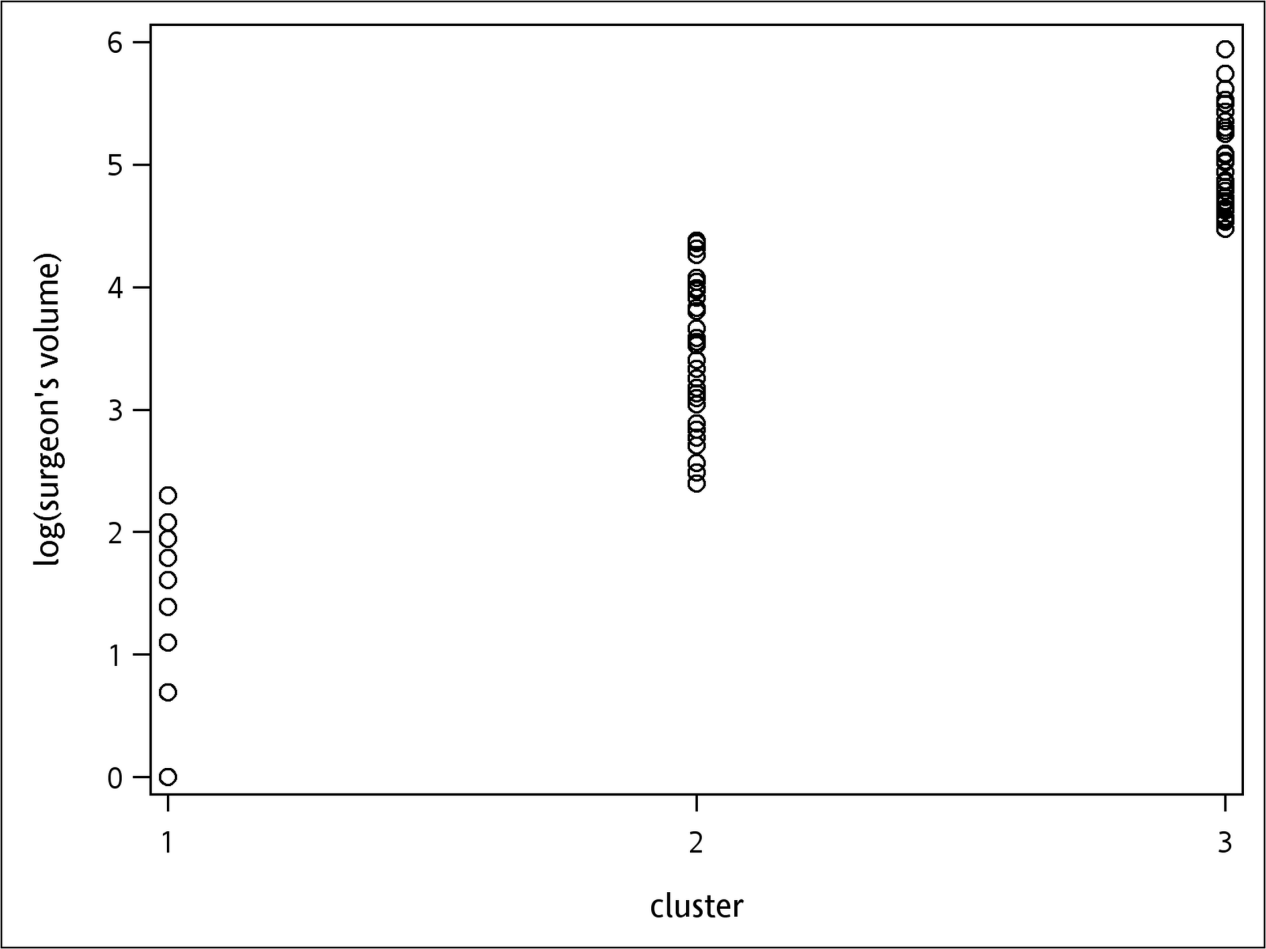
Scatter plot: surgeon cumulative operation volumes within the study period.

**Fig 3:**
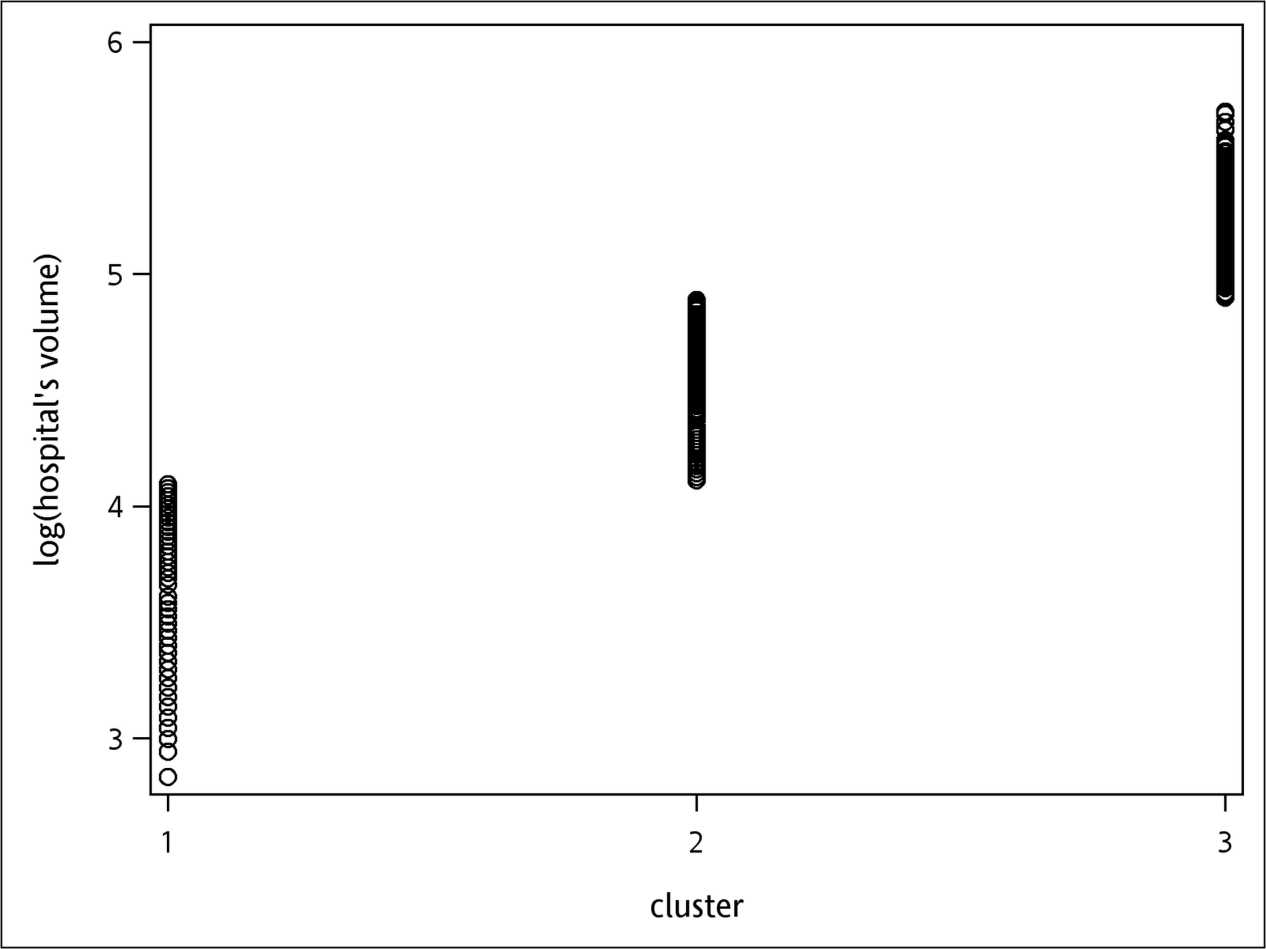
Scatter plot: hospital cumulative operation volumes in the previous one year before surgery.

**Fig 4:**
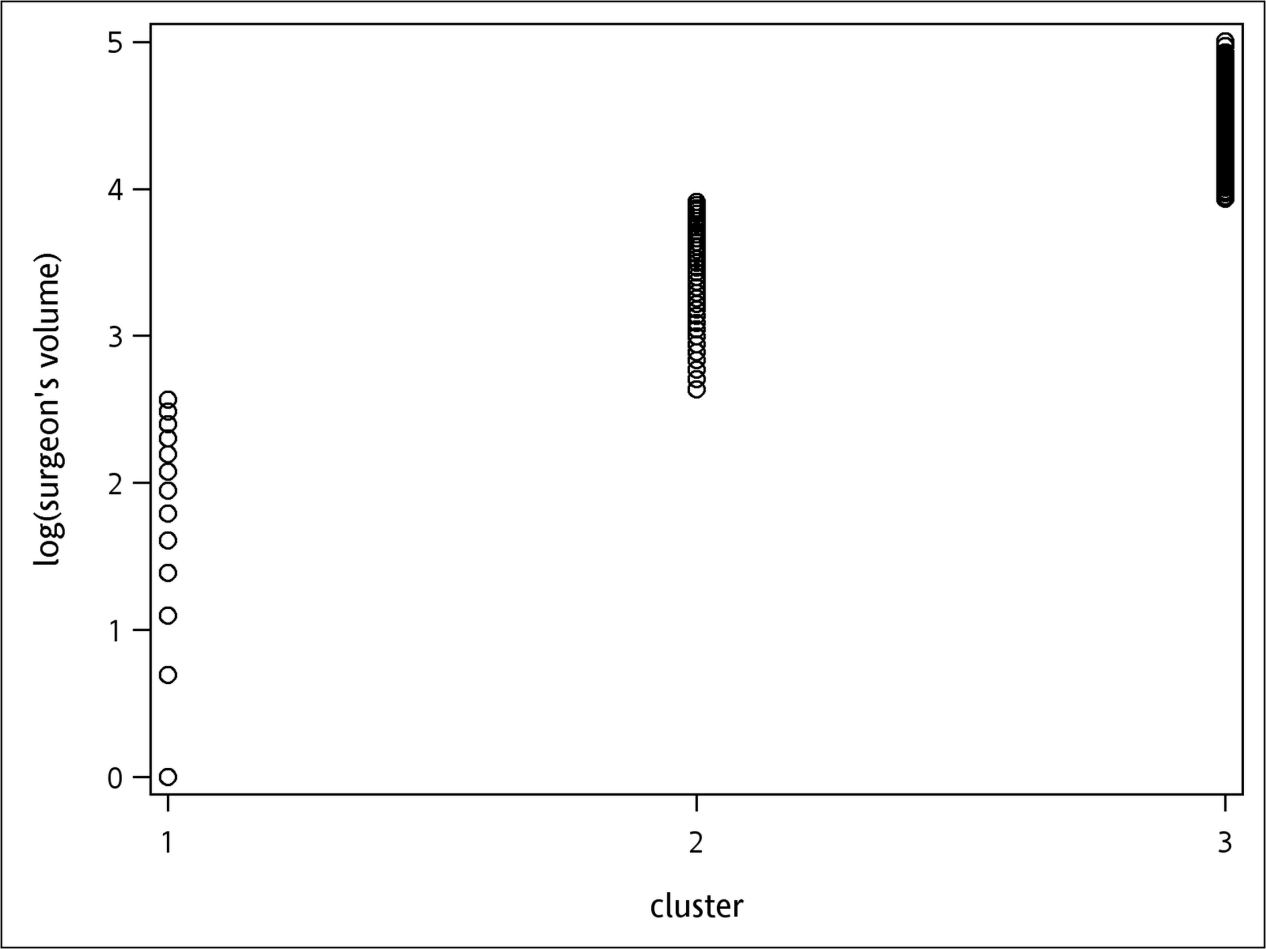
Scatter plot: surgeon cumulative operation volumes in the previous one year before surgery.

The cut-off values of hospital volumes were 5.21 and 5.69, and for surgeon’s volumes were 2.40 and 4.38 respectively, when cumulative operation volumes within the study period was used as the definition. Likewise, when cumulative operation volumes before each surgery was used as definition, the cut-off values were 4.11 and 4.89 for hospital volumes, and 2.64 and 3.91 for surgeon’s volumes. All cutoff values were transformed by logarithm. The results of k-means clustering are demonstrated in Figs 1–4. As the results show, the operation volumes were divided into three groups separately.

In addition to surgeon and hospital volumes and SSI, we collected patient-, surgeon-, and hospital-level data. Firstly, patient-level variables included age, gender, length of ICU stay, number of vessels obstructed that were involved in the surgical operation, and the presence of important underlying diseases (e.g. diabetes mellitus, chronic obstructive pulmonary disease (COPD), heart failure, renal failure and renal insufficiency, which were associated with SSI).[13] Secondly, the surgeon-level variables included age and gender. Thirdly, the hospital-level variables included hospital ownership and geographic location.

### Statistical analysis

All statistical analyses of volume-infection relationship were performed using *SAS* (version 9.2, SAS Institution Inc., Cary, NC, USA). In statistical testing, a two-sided *p* value ≤ 0.05 was considered statistically significant. The distributional properties of continuous variables were expressed by mean ± standard deviation (SD), whereas categorical variables were presented by frequency and percentage. In univariate analysis, the potential three-level predictors of SSI were examined using chi-square test or two-sample *t*-test as appropriate. Next, to account for the correlations within surgeon (level-2) and hospital (level-3), multivariate analysis was conducted by fitting mixed-effects logistic regression models to each patient’s data for estimating the effects of three-level predictors on the probability of post-operational SSI. Furthermore, subgroup analysis for comorbidities was also conducted.

## Results

Table 2 shows that there were 7,007 patients with CABG performed by 199 surgeons in 19 hospitals during 2006–2008 in Taiwan. The majority of patients were male (77.5%), and the mean age of patients was 65.3 years. The average ICU stay was 6.05 days, the mean level of number of vessels obstructed was around 1.6, while 51.8% of patients had diabetes mellitus, 33.3% had heart failure, 14.1% had renal failure and renal insufficiency, and 22.0% had COPD. Three hundred and two patients (4.31%) were identified as having the ICD-9-CM SSI codes. However, identification by the CART model only revealed 107 infection cases, and 94 cases were identified in both models. Most cases received CABG surgery by male surgeons, with a mean age of 45.0 years, and the surgeon’s average operation volumes within the study period was 151.64, while the average operation volumes before surgery was 52.18. More than half of the cases were performed with CABG in not-for-profit hospitals, and the hospitals’ average operation volumes within the study period was 473.60, while the average operation volumes before each surgery was 158.79. Moreover, most of patients received their surgeries by high-volume surgeons and hospitals, when k-means algorithm was used for categorization, regardless of which definition of operation volumes were used.

**Table 2:** Descriptive statistics (n = 7,007).

Variables	
**Patient-level**	
Age^†^	65.34(11.06)
Gender(male)^‡^	5,429(77.48)
Length of stay^†^	20.78(11.47)
Length of ICU stay^†^	6.05(6.29)
Number of vessels obstructed ^†^	1.64 (0.49)
Diabetes mellitus ^‡^	3,630(51.81)
Heart failure^‡^	2,331(33.27)
Renal failure and Renal insufficiency^‡^	987(14.09)
COPD^‡^	1,544(22.04)
SSI: identified by ICD-9-CM SSI code^‡^	302(4.31)
SSI: identified by the CART model ^‡^	107(1.53)
**Surgeon-level**	
Age^†^	45.00(7.73)
Gender(male)^†^	6,943(99.09)
Average cumulative volumes within study period^†^	151.64(97.77)
Average cumulative volumes in previous one year^†^	52.18(33.57)
**Hospital-Level**	
Ownership^‡^	
Public	3,282(46.84)
Not-for-profit	3,725(53.16)
Geographic location^‡^	
Highly urbanized areas	3,413(48.71)
Moderately urbanized areas	3,594(51.29)
Average cumulative volumes within study period^†^	473.60(185.87)
Average cumulative volumes in previous one year^†^	158.79(61.92)

^†^ mean (S.D)

^‡^N (%); SSI: surgical site infection; COPD: chronic obstructive pulmonary disease

Table 3 shows the results of multilevel mixed-effect models, with the SSIs being identified by ICD-9-CM codes, and the operation volumes defined as the cumulative volumes within the study period. The results of Model 1 (continuous) reveal that the surgeon’s volumes were negatively associated with SSIs, while hospital’s volumes were not associated with surgical site infection SSIs. Model 2 (quartile) suggests that low-volume surgeons had higher SSI risk (OR = 2.220, p-value = 0.022) than high-volume surgeons. There were also no associations between hospital’s operation volumes and SSIs. Model 3 (k-means) shows that the association did not exist between hospital’s/ surgeon’s volumes and SSIs.

**Table 3:** Results of Multilevel Analysis: Cumulative volumes within the study period and SSIs were identified by the ICD-9-CM codes.

	Model 1 (continuous)				Model 2 (quartile)				Model 3 (k-means)			
N = 7,007	OR	95%C.I.		p-value	OR	95%C.I.		p-value	OR	95%C.I.		p-value
		LCL	UCL			LCL	UCL			LCL	UCL	
Fixed effects												
**Hospital-level**												
Cumulative volumes	1.347	0.724	2.506	0.3224								
Cumulative volumes (Reference: high-volume)												
Low-volume					0.528	0.207	1.350	0.1667	0.672	0.217	2.079	0.4626
Medium-volume					0.541	0.226	1.300	0.1551	1.103	0.556	2.186	0.7639
**Surgeon-level**												
Cumulative volumes	0.844	0.743	0.958	0.0086								
Cumulative volumes (Reference: high-volume)												
Low-volume					2.220	1.133	4.350	0.0224	1.709	0.911	3.203	0.0922
Medium-volume					1.881	0.919	3.848	0.0806	1.385	0.936	2.048	0.1003

OR: odds ratio; C.I.: confidence interval; LCL: lower confidence limit; UCL: upper confidence limit

All models were adjusted by hospital ownership and geographic location, surgeon’s gender and age, patient’s age, gender, length of ICU stay, number of vessels obstructed and underlying diseases

Table 4 displays the results of multilevel mixed-effect models, in which the SSIs were identified by the CART model, and the operation volumes were also defined as the cumulative volumes within the study period. Model 1 again indicated a negative association between surgeon’s volumes and SSIs, and hospital’s volumes were not found to be associated with SSIs. In Model 2, the results showed that the relationship between hospital’s/ surgeon’s volumes and SSIs did not exist. In Model 3, results revealed low-volume surgeons had higher risk (OR = 1.691, p = 0.002) than high-volume surgeons.

**Table 4:** Results of Multilevel Analysis: Cumulative volumes within the study period and SSIs were identified by the CART model.

	Model 1 (continuous)				Model 2 (quartile)				Model 3 (k-means)			
N = 7,007	OR	95%C.I.		p-value	OR	95%C.I.		p-value	OR	95%C.I.		p-value
		LCL	UCL			LCL	UCL			LCL	UCL	
Fixed effects												
**Hospital-level**												
Cumulative volumes	0.937	0.369	2.381	0.8837								
Cumulative volumes (Reference: high-volume)												
Low-volume					1.208	0.279	5.241	0.7860	0.586	0.103	3.340	0.5208
Medium-volume					0.763	0.193	3.015	0.6787	1.623	0.616	4.270	0.3015
**Surgeon-level**												
Cumulative volumes	0.734	0.616	0.875	0.0006								
Cumulative volumes (Reference: high-volume)												
Low-volume					2.469	0.769	7.925	0.1216	3.774	1.691	8.424	0.0020
Medium-volume					1.986	0.608	6.485	0.2406	1.322	0.676	2.586	0.4038

OR: odds ratio; C.I.: confidence interval; LCL: lower confidence limit; UCL: upper confidence limit

All models were adjusted by hospital ownership and geographic location, surgeon’s gender and age, patient’s age, gender, length of ICU stay, number of vessels obstructed and underlying diseases

Table 5 displays the results of multilevel mixed-effect models, in which the SSIs were identified by ICD-9-CM codes, but the operation volumes were defined as the cumulative volume in the previous one year for each surgery. Model 1 also indicated a negative association between surgeon’s volumes and SSIs, and hospital’s volumes were not found to be associated with SSIs. In Model 2, the results showed that the relationship between hospital’s/ surgeon’s volumes and SSIs did not exist. In Model 3, results also revealed low-volume surgeons had higher risk (OR = 1.642, p = 0.040) than high-volume surgeons.

**Table 5:** Results of Multilevel Analysis: Cumulative volumes in the previous one year and SSIs were identified by the ICD-9-CM codes.

	Model 1 (continuous)				Model 2 (quartile)				Model 3 (k-means)			
N = 7,007	OR	95%C.I.		p-value	OR	95%C.I.		p-value	OR	95%C.I.		p-value
		LCL	UCL			LCL	UCL			LCL	UCL	
Fixed effects												
**Hospital-level**												
Cumulative volumes	1.434	0.834	2.466	0.1924								
Cumulative volumes (Reference: high-volume)												
Low-volume					0.678	0.303	1.516	0.2695	0.592	0.136	2.578	0.3394
Medium-volume					0.658	0.377	1.148	0.1111	0.973	0.428	2.214	0.9228
**Surgeon-level**												
Cumulative volumes	0.831	0.704	0.980	0.0280								
Cumulative volumes (Reference: high-volume)												
Low-volume					1.627	0.975	2.714	0.0615	1.642	1.024	2.633	0.0400
Medium-volume					1.484	0.947	2.325	0.0828	1.406	0.969	2.040	0.0712

OR: odds ratio; C.I.: confidence interval; LCL: lower confidence limit; UCL: upper confidence limit

All models were adjusted by hospital ownership and geographic location, surgeon’s gender and age, patient’s age, gender, length of ICU stay, number of vessels obstructed and underlying diseases

Table 6 displays the results of multilevel mixed-effect models, in which the SSIs were identified by the CART model, and the operation volumes were also defined as the cumulative volume in previous one year for each surgery. In Model 1, different to the above findings, there was no association between hospital’s/ surgeon’s volumes and SSIs. In Model 2, the results showed that the relationship between hospital’s/ surgeon’s volumes and SSIs did not exist. In Model 3, results also revealed low-volume surgeons had higher risk (OR = 1.163, p = 0.020) than high-volume surgeons.

**Table 6:** Results of Multilevel Analysis: Cumulative volumes in the previous one year and SSIs were identified by the CART model.

	Model 1 (continuous)				Model 2 (quartile)				Model 3 (k-means)			
N = 7,007	OR	95%C.I.		p-value	OR	95%C.I.		p-value	OR	95%C.I.		p-value
		LCL	UCL			LCL	UCL			LCL	UCL	
Fixed effects												
**Hospital-level**												
Cumulative volumes	0.988	0.949	1.028	0.8417								
Cumulative volumes (Reference: high-volume)												
Low-volume					1.205	0.338	4.295	0.7221	0.457	0.044	4.707	0.3638
Medium-volume					0.791	0.346	1.809	0.4982	1.439	0.481	4.307	0.3679
**Surgeon-level**												
Cumulative volumes	0.918	0.398	2.121	0.1279								
Cumulative volumes (Reference: high-volume)												
Low-volume					1.790	0.787	4.068	0.1571	2.454	1.163	5.181	0.0200
Medium-volume					1.160	0.556	2.421	0.6819	1.415	0.766	2.616	0.2578

OR: odds ratio; C.I.: confidence interval; LCL: lower confidence limit; UCL: upper confidence limit

All models were adjusted by hospital ownership and geographic location, surgeon’s gender and age, patient’s age, gender, length of ICU stay, number of vessels obstructed and underlying diseases

We further examined the associations of surgeon and hospital volumes with SSIs in stratification analyses by underlying diseases. When the operation volumes were defined as the cumulative operation volume within the study period, no relationships existed between hospital/ surgeon operation volumes and SSIs. (Table 7) However, when the operation volumes were defined as the cumulative operation volumes in the previous one year for each surgery, the results suggested that there was a negative association between surgeon volumes and SSIs in the diabetes group, except that the volumes were treated as continuous variable and the infection cases were identified by ICD-9 codes. In terms of hospital operation volumes, the association did not exist. (Table 8)

**Table 7:** Summarized Results of Subgroup Analysis for Underlying Diseases: Cumulative Operation Volumes within the Study Period.

	Model 1 (continuous)		Model 2 (quartile)(Ref = HV)		Model 3 (k-means) (Ref = HV)	
	surgeon	hospital	surgeon	hospital	surgeon	hospital
**SSIs were identified by ICD-9-CM SSI codes**						
Heart failure	X	X	X	X	X	X
Diabetes mellitus	X	X	X	X	X	X
Renal failure and Renal insufficiency	X	X	X	X	X	X
COPD	X	X	X	X	X	X
**SSIs were identified by CART**						
Heart failure	X	X	X	X	X	X
Diabetes mellitus	X	X	X	X	X	X
Renal failure and Renal insufficiency	X	X	X	X	X	X
COPD	X	X	X	X	X	X

X: no association; >: higher risk; SSI: surgical site infection; COPD: chronic obstructive pulmonary disease

LV: Low Volume; MV: Medium Volume; HV: High Volume

**Table 8:** Summarized Results of Subgroup Analysis for Underlying Diseases: Cumulative Volumes in the Previous One Year.

	Model 1 (continuous)		Model 2 (quartile)(Ref = HV)		Model 3 (k-means) (Ref = HV)	
	surgeon	hospital	surgeon	hospital	surgeon	hospital
**SSIs were identified by ICD-9-CM SSI codes**						
Heart failure	X	X	X	X	X	X
Diabetes mellitus	X	X	LV>HV	X	LV>HV	X
Renal failure and Renal insufficiency	X	X	X	X	X	X
COPD	X	X	X	X	X	X
**SSIs were identified by CART**						
Heart failure	X	X	X	X	X	X
Diabetes mellitus	LV>HV	X	LV>HV	X	LV>HV	X
Renal failure and Renal insufficiency	X	X	X	X	X	X
COPD	X	X	X	X	X	X

X: no association; >: higher risk; SSI: surgical site infection; COPD: chronic obstructive pulmonary disease

LV: Low Volume; MV: Medium Volume; HV: High Volume

## Discussion

No studies have evaluated how different service/ operation volumes definitions and categorization methods affect volume-infection relationships. Moreover, several studies have pointed out the inappropriateness of identifying infection cases using the ICD-9-CM codes in claims data. Given these reasons, this study adopted two approaches to identifying SSIs, two definitions of operation volumes, and three methods for categorizing operation volumes to examine the relationships between operation volumes and SSIs.

Our findings showed that the relationships between hospital volumes and SSIs did not exist, no matter which definitions, categorization mehods, or SSIs case identification approaches were used. On the contrary, the relationships between surgeon volumes and SSIs were not robust in our data. It might be affected by different definitions and categorization methods of operation volumes, and also by different SSI cases identification approaches. In summary, most of the models demonstrated that the low-volume surgeons had higher risk than high-volume surgeons, and they also showed the risks were similar between medium-volume and high-volume surgeons. However, why did surgeon volume relate to SSIs, but hospital volume did not? Except for those issues we were concerned about in this study, there are some disagreements in the literature. Such as “Does provider volume really represent quality of care?” [12, 35] Or “Is provider volume the only one predictor for outcome of care?” [35, 36] These issues are worthy of further discussion, but are out of the scope of this study.

Service/ operation volumes are treated as a proxy indicator for experiences; previous studies used it to examine whether practice makes perfect or not. But, except for provider’s experiences, SSIs are also impacted by many factors, such as environmental and clinical factors. Wu et al once used Taiwan 2001 NHI claims data to explore the relationship between provider CABG operation volumes and SSIs. [13] They found that hospital volumes had a greater effect than surgeon volumes and claimed that this may imply that hospital teamwork is more important than individual surgeon. However, our findings demonstrated that there was no relationship between hospital volumes and SSIs. Wu et al. adopted the cumulative operation volumes within the study period as the definition, and identified SSIs by ICD-9-CM codes. Except, there were two differences between our work and Wu et al., which were the length and year of the data; our data was longer and more updated than theirs. Moreover, it is worth noting that there was an outbreak of severe acute respiratory syndrome (SARS) in Taiwan in 2003, after which the hospital infection control system in Taiwan was reviewed and re-designed. Wu et al data was before SARS, so these efforts may also have improved the level of SSIs control in hospitals, leading to different findings in this study.

In addition, although most models revealed that there were negative relationships between surgeon’s volumes and surgical site infection, the relationships were not robust. The results varied between different definitions and categorization method of operation volumes, and between SSIs identification approaches. Researchers need to consider how to identify SSIs correctly, how to choose optimal cut-off values, and how to decide on which definition is appropriate.

Finally, the results of stratification analyses showed that low-volume surgeon had higher risk than high-volume surgeon in the diabetes mellitus group, when the cumulative operation in the previous one year before surgery was used as definition. A large number of studies have indicated diabetes mellitus is associated with a higher risk of SSIs, [37–39] and the findings of this study suggest that CABG patients with diabetes mellitus should be cared for by experienced surgeons.

A multilevel analysis was applied to manage the nested factors, and two definitions of operation volume along with three different operation volume categorization methods were adopted to examine the relationship between volume and SSIs under two kinds of SSIs identification approaches. Nevertheless, the study suffered from several major limitations. First, the accuracy of SSIs identification was still an issue. Although the performance of the CART model to identify CABG SSIs was better than ICD-9-CM codes in Taiwan NHI claims data, it did not reach the perfect scenario. The accuracy of SSIs identification was still a challenge in our work. The second limitation relates to unmeasured variables, such as length of stay before operation, infection condition, hair removal, clinical information (e.g. blood glucose level, causative microorganism), time-related information (e.g. the duration of operation), the environment, surgical skills, use of post-operative drains, number of operations involved, and surgical site and wound care, *etc*.[40] Furthermore, information about type (elective or urgent) and incision site for surgery was not available in the Taiwan NHI claims data.

## Conclusion

In conclusion, the findings of this study suggest that different definitions and categorization methods of operation volumes, and different SSIs identification approaches might lead to different findings, although surgeon volumes were more important than hospital volumes in exploring the relationships between CABG operation volumes and SSIs in Taiwan, but they were still not robust. Definitions and categorization methods of operation volumes, and correct identification of SSIs are important issues for future research.
